# Evaluation of the dose-related concentration approach in therapeutic drug monitoring of diuretics and β-blockers – drug classes with low adherence in antihypertensive therapy

**DOI:** 10.1038/s41598-019-52164-y

**Published:** 2019-10-30

**Authors:** Sabrina Ritscher, Milena Hoyer, Cora Wunder, Nicholas Obermüller, Stefan W. Toennes

**Affiliations:** 1 Institute of Legal Medicine, Department of Forensic Toxicology, University Hospital, Goethe University, Frankfurt/Main, Germany; 2 Department of Nephrology, Medical Clinic III, University Hospital, Goethe University, Frankfurt/Main, Germany

**Keywords:** Drug therapy, Hypertension, Laboratory techniques and procedures

## Abstract

Detection of antihypertensive drugs in biological samples is an important tool to assess the adherence of hypertensive patients. Urine and serum/plasma screenings based on qualitative results may lead to misinterpretations regarding drugs with a prolonged detectability. The aim of the present study was to develop a method that can be used for therapeutic drug monitoring (TDM) of antihypertensive drugs with focus on adherence assessment. Therefore, a method for quantification of four diuretics and four β-blockers using high-performance liquid chromatography-mass spectrometric analysis (LC-MS/MS) of combined acidic and basic serum extracts was developed and validated. The method was applied to 40 serum samples from 20 patients in a supervised medication setting (trough and peak serum samples). Literature data on therapeutic concentration ranges, as well as dose-related drug concentrations (calculated from data of pharmacokinetic studies) were used to evaluate adherence assessment criteria. Concentrations were measured for bisoprolol (n = 9 patients), metoprolol (n = 7), nebivolol (n = 1), canrenone (n = 2, metabolite of spironolactone), hydrochlorothiazide (n = 10) and torasemide (n = 8). The measured concentrations were within the therapeutic reference ranges, except for 24% of the samples (mainly β-blockers). In contrast, all measured concentrations were above the lower dose-related concentration (DRC), which appears superior in evaluating adherence. In conclusion, the quantitative analysis of antihypertensive drugs in serum samples and its evaluation on the basis of the individually calculated lower DRC is a promising tool to differentially assess adherence. This method could possibly detect a lack of adherence or other causes of insufficient therapy more reliably than qualitative methods.

## Introduction

Arterial hypertension is a major risk factor for cardiovascular disease worldwide and remains the leading cause of death in the western world^1^. In Europe approximately 4 million people die due to cardiovascular disorders every year^2^.

For therapy, drugs of five different classes are typically used (ACE-inhibitors, AT1 antagonists, β-blockers, calcium-channel blockers and diuretics). Non-adherence to a medication plan leads to poor blood pressure control in antihypertensive therapy^3^. This results in frequent visits to the doctor and increased hospital stays^4^. This in return causes greater health care cost^3^. Adherence rates appear to differ between the drug classes. In a meta-analysis by Kronish *et al*. of studies based on medication refill data, poorest adherence was found for diuretics (51%) and β-blockers (28.4%)^5^. This is supported by Gupta *et al*. revealing that the number of prescribed drugs alongside with the classes of antihypertensives, especially diuretics, are the main risk factors contributing to non-adherence^6^.

There are different methods of adherence assessment (i.e. self-reporting, prescription records, pill count, electronic monitoring systems and toxicological analyses)^7^, whereas urine and serum/plasma screenings are the only direct and specific methods^8,9^. However, the qualitative nature may lead to misclassification of adherence in case of excretion much longer than the dosing interval. A diagnostic advancement was achieved by implementing a quantitative method for 21 antihypertensive drugs in serum that was recently published by Gundersen *et al*.^10^. They set up a therapeutic drug monitoring (TDM) system and used pharmacokinetic data to establish calibration ranges to classify measured serum concentrations with respect to adherence.

For the present study a quantitative assay of the main diuretics and β-blockers according to German prescription data was developed and applied to serum of hypertensive patients with confirmed adherence. The concentrations were evaluated with respect to the diagnosis of non-adherence according to two concepts: published reference ranges and the calculated lower dose-related concentration as established for psychiatric TDM^11^.

## Materials and Methods

### Chemicals and reference standards

Reference substances furosemide, torasemide, atenolol, bisoprolol and metoprolol were obtained from Sigma-Aldrich GmbH (Steinheim, Germany). Hydrochlorothiazide (HCT), canrenone and nebivolol, as well as the deuterated internal standards (IS) ketamine-d_4_, haloperidol-d_4_, diazepam-d_5_, quetiapine-d_8_, oxazepam-d_5_ and methadone-d_9_ were purchased from LGC Standards GmbH (Wesel, Germany). HCT-d_2_ was obtained from Toronto Research Chemicals (North York, Canada).

Acetonitrile was obtained from Karl Roth GmbH (Karlsruhe, Germany) and ethyl acetate from AppliChem (Darmstadt, Germany). Further chemicals and solvents used were supplied by Sigma-Aldrich GmbH (Steinheim, Germany). All reagents and solvents were either of analytical or LC grade.

### Serum samples

Patients (15 males, 5 females) aged 32 to 81 (median 57) years treated with a constant dosing regimen of antihypertensive drugs at the nephrological ward at the University Hospital Frankfurt/Main (Germany) participated in this study. Two blood samples were collected in serum tubes in the morning. The first one shortly before (trough level) and the second approximately two hours after monitored oral administration of the medication (peak level). After centrifugation (2,000 × g for 10 min) the separated serum was stored at −20 C until analysis. For data evaluation information on hospital admission, medication regimen (dose, dosing interval, date of last dose adjustment, co-medication), times of drug intake and of blood sampling were collected. Hospital admission was documented to ensure that steady-state concentrations were reached by the time of blood sampling. The patients’ drug ingestion was monitored by the nurses. The study protocol was approved by the competent ethics committee of the Goethe University Frankfurt (reference no. 19/18) and the study was in accordance with the 1964 Helsinki declaration and its later amendments. Written informed consent was obtained from all individual participants included in the study.

### Sample preparation

An aliquot of 200 µl serum was transferred to a 2 ml polypropylene reaction tube and 1 ml ethyl acetate, 50 µl internal standard working solution (0.25 ng/µl ketamine-d_4_ and methadone-d_9_; 0.5 ng/µl haloperidol-d_4_, diazepam-d_5_, protriptyline-d_3_, quetiapine-d_8_ and oxazepam-d_5_ and 0.05 ng/µl HCT-d_2_), 10 µl of acetonitrile (or mixed standard solution or quality control mix, see below) and 50 µl formic acid (10%) were added. After mixing for 2 min and centrifugation at 13,000 × g for 10 min the organic phase was transferred to a silanized glass tube. Another 1 ml ethyl acetate and 50 µl of aqueous ammonia (25%) were added to the aqueous phase followed by mixing for 2 min, centrifugation and transferring to the glass tube. The combined extracts were evaporated at 25 °C with nitrogen using TurboVap LV (Biotage, Uppsala, Sweden). The dry residue was reconstituted with 100 µl of 0.1% formic acid/acetonitrile (80:20, v/v) and transferred to 300 µl glass vials of which 5 µl were analysed.

### Calibration standards and quality controls

Human drug-free serum for preparation of quality controls (QC) and calibration standards was provided by healthy volunteers. Stock solutions of furosemide and canrenone were prepared in acetonitrile, whereas torasemide, atenolol, bisoprolol, metoprolol, HCT and nebivolol were dissolved in methanol at concentrations of 1 mg/ml (drug) and kept refrigerated at −20 °C. These were used for preparation of mixed standard solutions and quality controls in acetonitrile. The calibration range was 0.1–20 ng/ml for nebivolol, 2.5–100 ng/ml for bisoprolol, 1–200 ng/ml for metoprolol, 5–1000 ng/ml for atenolol, canrenone, furosemide and HCT and 10–2000 ng/ml for torasemide. The corresponding QC levels (low, medium, high) are listed in Table 1.

### Liquid chromatography-mass spectrometry (LC-MS/MS)

The analysis was performed on an Agilent (Waldbronn, Germany) LC-MS/MS system consisting of a 1290 Infinity Liquid Chromatograph coupled via JetStream Electrospray Interface (ESI) to a G6460A Triple Quadrupole Mass Spectrometer. Extracts were kept at 20 °C on the autosampler and analytes were separated on a Kinetex® 2.6 µm XB-C18 100 Å LC column (30 × 2.1 mm) plus corresponding guard column from Phenomenex (Aschaffenburg, Germany) at 55 °C.

Gradient elution was performed at a flow rate of 0.4 ml/min using 0.01% formic acid containing 5 mM ammonium formate (A) and acetonitrile containing 0.1% formic acid (B). Gradient elution started with 5% B kept for 0.5 min, increased to 40% B during 2.7 min, maintained 0.3 min, enhanced to 50% B within 0.5 min, held for 0.5 min and increased during 1.5 min to 95% B, maintained 2 min and followed by re-equilibration for 2 min, resulting in a total run time of 8 min.

Source parameters were selected as follows: gas temperature 300 °C, gas flow 11 l/min, nebulizer 45 psi, sheath gas temperature 400 °C, sheath gas flow 12 l/min and capillary voltage 3500 V. Detection was performed in the dynamic multiple reaction monitoring mode (dMRM) according to the mass spectrometry parameters listed in Table 2. Data acquisition and evaluation was performed using Agilent MassHunter Software (version B.07.00). For identification of analytes a deviation of retention time of less than 0.05 min and a qualifier to quantifier ratio below 20% deviation compared to reference standards were required.

**Table 1:** Validation data: lower limit of quantification (LLOQ), limit of detection (LOD), intra- and inter-day precision, accuracy, recovery and matrix effects (±SD) measured using the given quality control levels.

Analyte	LLOQ (LOD) [ng/ml]	quality control [ng/ml]	intra-day precision [%]	inter-day precision [%]	accuracy [%]	recovery [%]	matrix effects ± SD [%]
Atenolol	0.027	62.5	7.1	7.1	4.2	50.0	92.1 ± 3.1
	(0.007)	250.0	3.9	6.8	5.9		
		625.0	5.3	5.9	−4.0	60.6	85.7 ± 2.7
Bisoprolol	0.006	18.75	5.2	7.4	2.9	85.8	91.8 ± 4.7
	(0.003)	62.5	7.9	7.9	−2.7		
		87.5	4.3	8.5	−3.7	93.6	99.9 ± 5.4
Metoprolol	0.011	12.5	7.1	7.1	1.5	58.3	100.4 ± 2.0
	(0.003)	50.0	7.0	7.0	4.7		
		125.0	6.0	6.0	−4.0	80.1	96.5 ± 4.3
Nebivolol	0.045	1.25	7.7	8.1	0.4	54.5	81.8 ± 7.9
	(0.018)	5.0	5.0	6.1	5.0		
		12.5	7.7	7.7	2.1	85.3	89.7 ± 7.1
Canrenone	0.023	62.5	5.4	6.8	2.5	59.2	92.0 ± 4.1
	(0.008)	250.0	4.3	4.6	6.8		
		625.0	5.0	5.0	−0.9	86.0	94.1 ± 6.8
Furosemide	0.093	62.5	4.6	8.7	6.0	60.0	109.3 ± 14.8
	(0.034)	250.0	6.3	7.2	5.9		
		625.0	6.2	8.0	1.0	83.6	94.1 ± 9.8
HCT	1.592^a^	187.5	4.0	4.0	−1.3	91.3	93.3 ± 9.2
	(0.525)^a^	625.0	3.4	3.4	0.6		
		875.0	2.5	2.7	0.6	92.5	101.2 ± 2.3
Torasemide	0.009	187.5	4.4	8.4	−8.9	66.1	101.7 ± 5.7
	(0.016)	625.0	8.4	8.4	−2.5		
		875.0	5.1	5.8	−3.7	94.5	93.0 ± 6.5

^a^Determined according to ICH guidelines^33^.

### Method validation

The method was validated according to current guidelines^12^. Statistical evaluation was performed with Valistat 2.0 Software (Arvecon GmbH, Walldorf, Germany).

In order to find appropriate internal standards for the analytes, 41 deuterated medical and illicit drugs were tested. Thus, a broad spectrum of substances with different chemical and chromatographic properties was evaluated. Internal standards were assigned regarding linearity and compensation of matrix effects. Retention time was the decisive factor if deuterated substances yield similar results.

Matrix effects were evaluated by comparing peak areas of spiked extracts with those of standard solutions and recovery by comparing spiked matrix samples to spiked extracts. Both were determined in low and high quality control samples. Each QC sample was measured six times using blank serum samples of different donors.

Selectivity was assessed with human serum samples from eight different drug-free volunteers. Six samples were prepared without (blank samples) and another two by adding internal standard solution (zero samples). To show the absence of interferences serum samples with exogenous substances including typical therapeutic drugs and metabolites, as well as a range of psychoactive substances were analyzed. Sensitivity was assessed by analysing five calibrator concentrations evenly spaced in the range of the expected limit of detection (LOD) and lower limit of quantification (LLOQ) as previously described^13^.

Evaluation of linearity was done by six-fold determination in one sequence of seven calibration levels evenly distributed across the calibration range. The calibration was checked for outliers (Grubbs test), homogeneity (Cochran test) and linearity (Mandel test).

For verification of accuracy and precision homogenous pools of low, medium and high quality control samples (relative to calibration range) were prepared by spiking blank matrix and dividing into aliquots. Thereafter two quality controls of each concentration level were measured on eight different days. Results were tested for accuracy (bias ≤15%) and intra- and inter-day precision (relative standard deviation ≤15%).

The analytes are sufficiently stable during long-term storage and during freeze-thaw cycles^10,14–16^. Stability of extracted analytes was tested under autosampler conditions for 72 h. The decrease in concentration of low and high QC samples was checked by repeated injection of an aliquot.

### Evaluation of concentrations

The measured concentrations were evaluated by comparison with therapeutic reference ranges as well as with lower limits of calculated dose-related concentrations. Therapeutic reference ranges, indicating therapeutic efficacy and acceptable tolerability, were retrieved from the list of Schulz *et al*.^17^ and Repetto *et al*.^18^. If the literature data did not match, the larger reference range was selected for evaluation (Table 3).

The approach to use dose-related concentrations (DRC) consists of comparing measured concentrations with trough serum drug concentrations calculated individually for each patient. To simplify the calculation of expected serum levels, first a factor (DRC factor) was calculated depending on the dosing interval (τ) which is equal to the blood sampling time before the next dose (∆t, 12 or 24 h for both parameters). The necessary pharmacokinetic parameters were retrieved from pharmacokinetic studies on patients without comorbidities, co-medication or genetic abnormalities after oral administration (Table 3): bioavailability f, total body clearance CL_t_, elimination rate constant k_e_.

1 $$DRC\,factor=\frac{f}{24\,\ast \,C{L}_{t}}\,\ast \,\frac{\tau \,\ast \,{k}_{e}}{1-{e}^{-{k}_{e}\ast \tau }}\ast {e}^{-{k}_{e}\ast \Delta t}[\frac{ng}{ml}/mg]$$

To cover inter-individual variabilities, the standard deviation (SD) of the apparent total clearance (CL_t_/f) as correlate of elimination was incorporated in the calculation (lower DRC factor, based on the concept of Hiemke *et al*.^11^) in a second step.

2 $$lower\,DRC\,factor=DRC\,factor-(\frac{SD}{C{L}_{t}/f}\,\ast \,DRC\,factor)\,[\frac{ng}{ml}/mg]$$

For each patient the expected trough serum concentration (lower DRC in ng/ml) was calculated by multiplication of the total daily dose in mg with the lower DRC factor. This lower limit of the dose-related concentration was used as a cut-off to evaluate concentrations with regard to adherence assessment.

## Results

### Method validation

**Figure 1:**
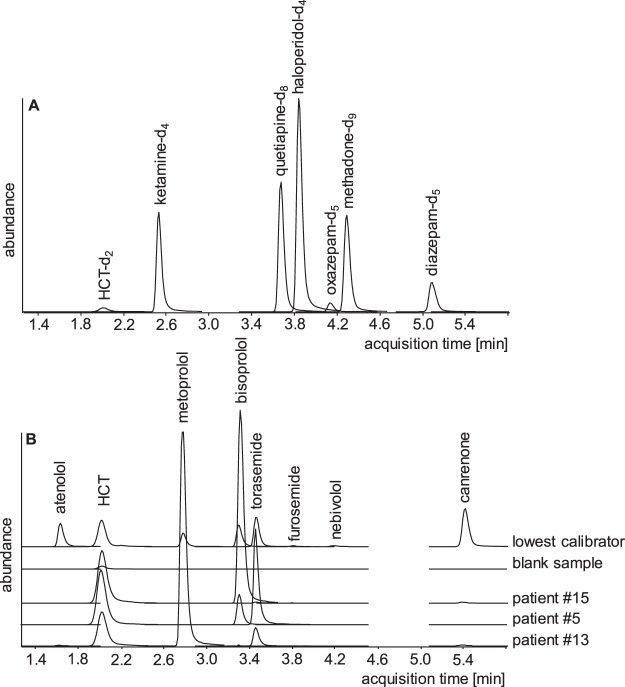
Representative extracted ion chromatograms of internal standards are given in (**A**) and of all analytes in the lowest calibrator, a blank sample, and trough serum samples of patient #15 on HCT (140.5 ng/ml) and bisoprolol (30.2 ng/ml), patient #5 on HCT (139.5 ng/ml), bisoprolol (8.7 ng/ml) and torasemide (439.6 ng/ml) and patient #13 on HCT (108.4 ng/ml), metoprolol (48.5 ng/ml) and torasemide (1752.9 ng/ml) in (**B**), all signals in equal scale.

This method based on a two-step liquid liquid-extraction at acidic and basic pH was validated for quantification of four β-blockers and four diuretics. Blank and zero serum samples showed no significant interference in terms of endogenous substances at retention times of analytes or internal standards except that a signal at the retention time of HCT was detected (c.f. chromatogram of a blank sample in Fig. 1) which obviously resulted from non-deuterated HCT present in the HCT-d_2_. In this case the LOD and LLOQ were determined based on the standard deviation of the response and the slope according to ICH guidelines (Table 1). Apart from this, neither the spiked nor the analysed samples from patients exhibited signals from other drugs.

**Table 2:** Mass spectrometry parameters for the detection of β-blockers and diuretics using LC-MS/MS operated in dynamic MRM mode with two transitions for analytes and one for the corresponding internal standard. Retention times, MRM transitions and collision energies (CE) were as follows.

Analyte	Retention Time [min]	Precursor Ion [m/z]	Quantifier [m/z] (CE [eV])	Qualifier [m/z] (CE [eV])	Internal standard
Atenolol	1.62	267.2	145.0 (24)	74.1 (20)	Ketamine-d4
HCT	2.04	295.9	268.9 (12)	78.0 (32)	HCT-d_2_
Metoprolol	2.79	268.2	74.1 (20)	116.1 (16)	Ketamine-d4
Bisoprolol	3.27	326.2	116.1 (16)	74.1 (24)	Haloperidol-d_4_
Torasemide	3.47	349.1	264.0 (12)	183.2 (32)	Methadone-d_9_
Furosemide	3.82	329.0	205.0 (16)	78.0 (4)	Oxazepam-d_5_
Nebivolol	4.14	406.2	151.0 (32)	103.1 (72)	Quetiapine-d_8_
Canrenone	5.44	341.2	107.1 (36)	91.1 (70)	Diazepam-d_5_
**Internal Standard**					
HCT-d_2_	2.06	298.0	270.0 (12)		
Ketamine-d_4_	2.53	242.1	129.0 (28)		
Quetiapine-d_8_	3.67	392.2	258.1 (20)		
Haloperidol-d_4_	3.84	380.2	169.1 (20)		
Oxazepam-d_5_	4.19	292.1	246.0 (20)		
Methadone-d_9_	4.28	319.3	268.1 (8)		
Diazepam-d_5_	5.12	290.1	198.1 (32)		

Analyte signals were not affected by ion suppression or enhancement. The limit of detection, lower limit of quantification, intra- and inter-day precision, accuracy and recovery as part of the validation procedure are summarized in Table 1. The LLOQs were below expected serum concentrations. The requirements of the Grubbs test (95% significance level), Cochran test (99% significance) and Mandel test (99% significance) were fulfilled and a non-weighted calibration model excluding the origin was used. The calibration curves covered therapeutic ranges and were linear with regression coefficients of at least 0.999. Intra- and inter-day precisions were less than 8.7%, accuracies less than 8.9% (mostly <5.0%) and recoveries higher than 50%. The concentration of the extracted analytes decreased less than 25% during 72 hours of measurement, except of torasemide (48 h), nebivolol (24 h) and canrenone (16 h). Therefore, analysis was always completed within half a day. Representative chromatograms are shown in Fig. 1.

### Serum samples

In this study serum of 20 patients on β-blockers and/or diuretics were evaluated. All expected drugs could be quantitated (Table 4) where the trough levels before medication were of special interest. As expected, a marked increase in serum concentrations was observed in the second serum samples representing the time around peak concentrations with a few exceptions (HCT in #16, all metoprolol concentrations).

Reference data on therapeutic plasma levels were used from various sources^17,18^ (Table 3) with the exception of torasemide for which no data was available. A high proportion of values were within the expected therapeutic reference ranges (75.9% of all determined concentrations). None exceeded the higher limit, but canrenone concentrations were mostly (75.0%) below the reported range, as well as 42.9% of metoprolol serum levels. For bisoprolol trough and peak concentrations of patient #5 were both below the therapeutic range (<10 ng/ml^17^) as were the trough samples of patients #2 and #20. In one case a HCT concentration (patient #20, trough) was lower than expected (<40 ng/ml^17^).

**Table 3:** The data from pharmacokinetic studies refer to healthy volunteers (n = total number of volunteers) with data on bioavailability (f), dosing interval (τ), apparent total clearance (CL_t_/f) and its standard deviation (SD), average elimination half-life (t_½_), the mean dose related concentration (DRC) factor with its lower limit for two time intervals between last dose and blood sampling (Δt). The last column cites the therapeutic reference range as retrieved from Schulz *et al*.^17^.

Drug	n	f	τ [h]	CL_t_/f [ml/min]	SD [ml/min]	t_1/2_ [h]	Δt [h]	DRC factor [ng/ml/mg]	lower DRC factor [ng/ml/mg]	reference	therapeutic range [ng/ml]^17^
Atenolol	30	0.55	24 12	178.3^a^	38.4	6.1	24 12	0.404 0.998	0.356 0.880	^28, 34, 35^	200–450
Bisoprolol	32	0.88	24 12	337.0^a^	76.2	14.7	24 12	1.111 1.533	0.860 1.186	^36, 37^	10–100
Metoprolol tartrate	10	0.55	24 12	1454.6^a^	181.8	4.1	24 12	0.034 0.147	0.030 0.128	^14, 38^	20–600^18^
Metoprolol succinate^b^	24	0.45	24 12	2857.2^a^	478.0	3.0	24 12	0.005 0.045	0.004 0.037	^39, 40^	
Nebivolol	69	0.12 EM^d^	24 12	7166.7^a^	1611.1	10.3	24 12	0.039 0.063	0.030 0.049	^26, 41, 42^	<20
		0.96 PM^d^	24 12	307.3^a^	n/a	33.0	24 12	1.738 1.987	n/a n/a		
Canrenone^c^	25	0.25	24 12	1208.0^a^	520.0	14.9	24 12	0.312 0.429	0.178 0.244	^43, 44^	100–250
Furosemide	11	0.47	24 12	589.5^a^	150.0	1.9	24 12	0.002 0.066	0.001 0.049	^45^	2000–5000
HCT	58	0.65	24 12	569.4^a^	172.5	10.6	24 12	0.501 0.802	0.349 0.559	^14, 26^	40–2000
Torasemide	37	0.79	24 12	43.0	9.8	3.7	24 12	0.819 4.287	0.632 3.310	^46– 48^	n/a

^a^Clearance is calculated by dividing the dose by the AUC.

^b^Sustained-release formulation.

^c^Administered as spironolactone.

^d^Genetic polymorphism: data for extensive metabolizers (EM) was used in the present study which differ markedly from those for poor metabolizers (PM).

In addition to evaluation of concentrations with regard to published reference ranges, the data was also compared with the expected lower limit of the trough serum concentration (lower DRC). This value was individually calculated on the basis of the patient’s drug dose and the drug’s lower DRC factor (Table 3). The lower DRC includes a diminution by one standard deviation of the apparent total clearance to reflect interindividual variations in excretion. All serum concentrations (trough and peak) of bisoprolol, nebivolol, metoprolol, canrenone, HCT, and torasemide were above these calculated limits.

## Discussion

Hypertension is the leading factor for cardiovascular morbidity and mortality^19,20^. Even though there are several pharmacological treatment options, effective high blood pressure management is an ongoing real concern. Since hypertension causes only few symptoms, there is a risk of poor adherence to drugs with unpleasant side effects. Patients not complying with their medication scheme (non-adherence) risk exhibiting a treatment resistant hypertension (TRH). Non-adherence is not easy to diagnose with current methods. Assessment of adherence by direct methods such as toxicological urine or blood analysis is available only occasionally. Based on our detailed previous experience with antihypertensive drug testing in urine^8,21^ this methodology is well suited to detect non-adherence, but still has some limitations. One problem is, that a few substances are excreted mainly as metabolites (especially dihydropyridine derivatives^22,23^) and a failure in detection of the drug might lead to misclassification as non-adherent. On the other hand, substances with prolonged excretion may be detectable despite poor adherence (e.g. HCT^24^). As a potential solution it was hypotheticized whether quantitative assays of the drugs in blood would reflect adherence more precisely. In addition, other causes of TRH like malabsorption or individual differences in excretion could be diagnosed more accurately with such an approach.

As a first step a quantitative chromatographic-mass spectrometric target compound analysis procedure was developed and validated. The assay focused on the mainly prescribed diuretics and β-blockers in Germany according to the annual Drug Prescription Report^25^. In a pilot study this method was applied to serum samples obtained from patients in the University Hospital Frankfurt/Main (Germany) with confirmed medication adherence. Results were evaluated with regard to two aspects: (1) does the analytical method yield concentrations that are in accordance with results from published studies and (2) can adherence be confirmed? For this latter aspect the measured concentrations were compared with published reference ranges and with individually calculated cut-off concentrations on the basis of the applied doses.

### Concentrations

Of the 20 patients 9 were treated with bisoprolol, which was confirmed in concentrations (trough and peak) of 3.3 to 53.8 ng/ml, 7 with metoprolol (5.8 to 110.8 ng/ml), one with nebivolol (0.36 and 1.08 ng/ml), 10 with HCT (15.5 to 606.3 ng/ml), 8 with torasemide (17.6 to 1829.2 ng/ml) and 2 with canrenone (25.5 to 100.4 ng/ml, active metabolite of administered spironolactone). The measured concentration ranges are in accordance with those found in samples of a routine TDM^10^ for bisoprolol (8.14–44.6 ng/ml), metoprolol (3.74–267 ng/ml), canrenone (14.0–91.2 ng/ml) and HCT (7.44–298 ng/ml). However, no data on daily doses or times of blood sampling was provided for a more detailed comparison. The larger concentration ranges in the present data are in agreement with the sampling scheme targeting the minimal (trough) and maximal (peak) concentrations in the patients. The present results also match reported serum concentrations of nebivolol and torasemide^26,27^. Rather high peak and trough concentrations of HCT were found for two patients (#8 and #18, Table 4) which is in agreement with a mean of 673.17 ng/ml that has been reported for elderly hypertensive patients on a daily dose of 25 mg^28^. Therefore, the concentrations measured in the present study are in agreement with published data from patients taking β-blockers and/or diuretics.

**Table 4:** Concentrations of β-blockers and diuretics in serum samples of patients shortly before and about 2 h after observed ingestion (trough/peak). Concentrations below published therapeutic reference ranges are indicated by “↓” (except for torasemide due to missing reference data), no concentrations below the lower DRC (lower DRC factor * daily dose) were observed.

Patient #	Drug	Daily dose (single) [mg]	lower DRC [ng/ml]	Bisoprolol [ng/ml]	Metoprolol [ng/ml]	Nebivolol [ng/ml]	HCT [ng/ml]	Torasemide [ng/ml]	Canrenone [ng/ml]
1	Metoprolol	50 (25)	1.9		6.6↓/7.3↓				
2	Bisoprolol	2.5	2.2	8.9↓/19.2					
3	Bisoprolol	5 (2.5)	5.9	15.8/16.5					
	HCT	12.5	4.4				100.5/200.2		
4	HCT	12.5	4.4				159.8/264.6		
	Metoprolol	200 (100)	7.4		38.7/42.0				
	Torasemide	20	12.6					50.9/1779.2	
5	Bisoprolol	1.25	1.1	3.3↓/8.7↓					
	HCT	12.5	4.4				44.2/139.5		
	Torasemide	5	3.2					91.3/439.6	
6	Metoprolol	100 (50)	3.7		12.6↓/10.7↓				
7	Metoprolol	47.5	0.2		5.8↓/7.3↓				
8	HCT	25	8.7				286.6/542.5		
9	Bisoprolol	5 (2.5)	5.9	16.4/23.1					
	Torasemide	10	6.3					320.4/1592.4	
10	Spironolactone	25	4.5						47.5↓/100.4
	Torasemide	5	3.2					371.5/1829.2	
11	Bisoprolol	2.5 (1.25)	3.0	15.4/10.9					
	Torasemide	5	3.2					35.1/86.2	
12	Nebivolol	5	0.2			0.4/1.1			
13	HCT	25	8.7				75.2/108.4		
	Metoprolol	190 (95)	7.0		49.4/48.5				
	Torasemide	20	12.6					24.8/1752.9	
14	Metoprolol	200 (100)	7.4		110.8/92.8				
	Torasemide	5	3.2					17.6/1277.9	
15	Bisoprolol	5	4.3	12.9/30.2					
	HCT	12.5	4.4				81.0/140.5		
16	HCT	12.5	4.4				69.5/60.8		
17	Bisoprolol	10 (5)	11.9	21.5/53.8					
18	Spironolactone	25	4.5						25.5↓/44.7↓
	HCT	25	8.7				317.9/606.3		
	Metoprolol	200 (100)	7.4		30.0/24.5				
19	Bisoprolol	10 (5)	11.9	41.1/53.8					
	HCT	12.5	4.4				113.9/167.5		
	Torasemide	20	12.6					39.6/1570.4	
20	Bisoprolol	10	8.6	9.8↓/25.1					
	HCT	25	8.7				15.5↓/96.4		

### Assessment of adherence on the basis of serum concentrations

Adherence assessment is an important part of the diagnosis of treatment resistant hypertension. Several methods have been published to assess patients’ adherence^29^. However, so far none of those employed quantitative data in combination with cut-off values.

Adherence rates appear to differ between the classes of antihypertensive medications. Especially low adherence was found for diuretics and β-blockers based on medication refill data^5^ which contrasts data from studies using urine or plasma analysis, where these classes were among those with the highest adherence rates^8,30^. In the evaluation of this discrepancy it must be taken into account that toxicological analyses are qualitative in nature and may still be positive even if some time passed since the last drug ingestion. Therefore, it appears necessary to extend toxicological analysis by a quantitative feature and evaluate concentrations in terms of pharmacological activity.

Data on the therapeutic concentration ranges have been reported only for five of the six drugs assayed, torasemide concentrations were therefore excluded from evaluation. None of the concentrations measured exceeded the upper therapeutic limits, but 14 of the total of 58 values (24.1%, 31.0% of the 29 trough values) fell below the lower limit of the concentration range considered therapeutic^17,18^. This affected mainly the β-blockers bisoprolol (especially low doses) and metoprolol, as well as the spironolactone metabolite canrenone (Table 4) and would lead to classification as non-adherent. Since in all cases drug ingestion was monitored this renders the published data as not reliable to differentiate drug ingestion by comparison with the lower limit of the therapeutic reference range. Obviously, reference ranges reflect pharmacologically effective concentrations but cannot be used to evaluate adherence.

For therapeutic drug monitoring (TDM) of antidepressants and neuroleptics this has been improved by Hiemke *et al*.^11^. Expected trough concentrations under steady-state conditions were calculated using a function described by Gex-Fabry *et al*.^31^ taking into account dose and dosing interval. In the present study this established concept was applied to evaluate concentrations of antihypertensive drugs. Therefore, lower limits of expected therapeutic concentrations were calculated for different dosing regimens. This based on the concept of Hiemke *et al*.^11^ for neuropsychopharmacology where complex dosing regimens were simplified by calculating the total daily dose with a hypothetical dosing interval of 24 h. In the present context this was extended by inclusion of the dosing interval as different doses during a day are rather rare in antihypertensive therapy. This leads to a more appropriate estimation of trough concentrations which are used as cut-offs and are thus more reliable for differentiation of adherence state.

All measured values were above the calculated minimum concentrations expected for the respective dosage schemes. The superiority of this evaluation concept has also been shown for TDM in neuropsychopharmacology^11^. However, limitations of this concept should not be disregarded. For metoprolol, it was striking that serum concentrations of each patient showed hardly any variation from trough to peak. From the patients records it was retrieved that all participants received metoprolol succinate as a sustained-release formulation. From this, a much longer time to maximal concentrations (t_max_) and smaller peak-trough fluctuations in serum concentrations are expected. Due to the continuous release over 20 hours, a postabsorptive phase, as with the other drugs, does not occur. Since the DRC concept relies on a forecast of the elimination which is different with sustained-release formulations the lower DRC was calculated using pharmacokinetic parameters from appropriate studies (Table 3). Therefore, this concept still allows comparison with measured serum concentrations but an underestimation cannot be excluded. Another limitation that arises in qualitative as well as quantitative methods are substances with low plasma levels and short half-lives. In the present study for instance, doses of furosemide once daily may result in trough concentrations which are very close to the LOD. It is therefore recommended to critically consider the applicability of the quantitative method for adherence assessment in such cases. A peculiarity which remains problematic is that adherence varies over time^32^ and the rare ingestion of drugs, especially prior to a doctor’s visit (white coat adherence), results in therapeutic serum concentrations which may lead to the false assumption of continuous adherence.

## Conclusion

The present proof-of-concept study was performed to evaluate an improved strategy for the assessment of adherence based on quantitative serum drug concentrations. The results with patients on supervised medication adherence show, that established ranges of therapeutic concentrations as far as they are available are not applicable to multi-drug regimens as used for treatment of hypertension. The calculation of lower limits of dose-related concentrations can be used as cut-off values. Concentrations beneath these thresholds may indicate non-adherence or deviations in pharmacokinetics (e.g. malabsorption or rapid drug elimination) which could be used for adaptation of the dosage scheme.

The superiority of evaluating quantitative serum results for assessment of adherence will be investigated in comparison to qualitative results in urine analysis in a study with outpatients without controlled adherence. In addition, the application of this approach to a wider range of antihypertensive drugs is in progress.
